# Improved Productivity of Neutral Lipids in *Chlorella* sp. A2 by Minimal Nitrogen Supply

**DOI:** 10.3389/fmicb.2016.00557

**Published:** 2016-04-21

**Authors:** Junying Zhu, Weixian Chen, Hui Chen, Xin Zhang, Chenliu He, Junfeng Rong, Qiang Wang

**Affiliations:** ^1^SINOPEC Research Institute of Petroleum ProcessingBeijing, China; ^2^Key Laboratory of Algal Biology, Institute of Hydrobiology, Chinese Academy of SciencesWuhan, China; ^3^University of Chinese Academy of SciencesBeijing, China

**Keywords:** algal cultivation, biofuel feedstock, biomass, *Chlorella* sp. A2, minimal nitrogen supply, lipid

## Abstract

Nitrogen starvation is an efficient environmental pressure for increasing lipid accumulation in microalgae, but it could also significantly lower the biomass productivity, resulting in lower lipid productivity. In this study, green alga *Chlorella* sp. A2 was cultivated by using a minimal nitrogen supply strategy under both laboratory and outdoor cultivation conditions to evaluate biomass accumulation and lipid production. Results showed that minimal nitrogen supply could promote neutral lipid accumulation of *Chlorella* sp. A2 without a significant negative effect on cell growth. In laboratory cultivation mode, alga cells cultured with 18 mg L^−1^ d^−1^ urea addition could generate 74 and 416% (w/w) more neutral lipid productivity than cells cultured with regular BG11 and nitrogen starvation media, respectively. In outdoor cultivation mode, lipid productivity of cells cultured with 18 mg L^−1^ d^−1^ urea addition is approximately 10 and 88% higher than the one with regular BG11 and nitrogen starvation media, respectively. Notably, the results of photosynthetic analysis clarified that minimal nitrogen supply reduced the loss of photosynthetic capacity to keep CO_2_ fixation during photosynthesis for biomass production. The minimal nitrogen supply strategy for microalgae cultivation could promote neutral lipid accumulation without a significant negative effect on cell growth, resulting in a significant improvement in the lipid productivity.

## Introduction

In the past few decades, the extensive utilization and irreversible depletion of fossil fuels has led to global climate change, environmental pollution, health problems, and an energy crisis (Amaro et al., 2011; Chen et al., 2016). Biofuel, as a new, alternative, clean, and sustainable energy source, has attracted great interest from researchers, local governments, and international traders (Lam and Lee, 2012). A variety of biolipids can be used to produce biodiesel, which is one of the most commonly used biofuel, and vegetable oils, such as soybean, sunflower, rapeseed, and palm oil, are renewable and potentially inexhaustible sources of energy with an energy content close to that of diesel fuel (Demirbas, 2011; Zhang et al., 2014). However, extensive use of vegetable oils may cause significant problems, for example, abundance of resource applied to produce fuel will cause starvation in developing countries, and it is important that productive and cultivated land should be used for food instead of fuel production (Paiva and Wolde-Georgis, 2010). One possible alternative, non-food, source of biological material for biofuel production is microalgae (Day et al., 1999; Qiao et al., 2015), which can be feedstock for biofuel production via photosynthesis by harvesting solar energy and fixing CO_2_ and convert it into biolipids (Razeghifard, 2013; Chen et al., 2015b). Moreover, microalgae are capable of rapid growth under a wide range of culture conditions and more photosynthetically efficient than higher plants (John Pirt, 1986). Thus, microalgae are now considered to be one of the most promising types of feedstock for making biodiesel (Mata et al., 2010; Chen et al., 2014).

The green microalga *Chlorella* (Chlorophyta) can grow photoautotrophically, mixotrophically, or heterotrophically under various culture conditions, with high biomass yield. The oil content in some species of *Chlorella* varies from about 14 to 63% of dry weight, and the fatty acid composition has been reported to range from C-14:0 to C-20:0 (O'Grady and Morgan, 2011). Considering the above advantages, *Chlorella* would appear to be a good material for biodiesel production.

Microalgal lipid accumulation is influenced by culture conditions. Previous studies have indicated that the lipid content of some microalgae can be increased by means of nitrogen (N) starvation (Illman et al., 2000; Takagi et al., 2000; Rodolfi et al., 2009; Chen et al., 2015a). The general theory is that when there is insufficient N for the protein synthesis required for growth, excess carbon from photosynthesis is channeled into storage molecules e.g., triglycerides or starch (Scott et al., 2010). However, although N starvation can increase the lipid content, it is not conducive for biomass accumulation or lipid productivity of microalgae (Vona et al., 1999).

In our previous study (Zhang et al., 2013), it was reported that N starvation resulted in significant neutral lipid accumulation in *Chlorella* cells, but in the meantime decreased photosynthetic rate, oxygen evolution, respiration rate, and photochemistry efficiency, as well as increasing damage to the Photosystem II (PSII), were observed. Therefore, the lipid productivity of microalgae was not significantly enhanced under N starvation condition.

In this study, a minimal nitrogen supply strategy was used in the cultivation of *Chlorella* sp. A2 (Hu et al., 2008) (an oil-producing microalgae isolated from the wild) for improving neutral lipid productivity. Results demonstrated that the minimal nitrogen supply strategy for microalgae cultivation could promote neutral lipid accumulation without a significant negative effect on cell growth, resulting in a significant improvement in the neutral lipid productivity.

## Materials and methods

### Microalgal strain and culture condition

*Chlorella* sp. A2, also known as *Chlorella* sp. NJ-18, was isolated from the wild and provided by Hu et al. (2008) and maintained in regular BG11 medium in the light. The N-sufficient medium (N+) used was full-strength BG11 medium (K_2_HPO_4_·3H_2_O, 0.04 g L^−1^, MgSO_4_· 7H_2_O, 0.075 g L^−1^, CaCl_2_, 0.027 g L^−1^, Citric Acid, 0.006 g L^−1^, Ammonium ferric citrate, 0.006 g L^−1^, EDTANa_2_, 0.001 g L^−1^, Na_2_CO_3_, 0.001 g L^−1^, NaNO_3_, 1.5 g L^−1^, H_3_BO_3_, 2.86 mg L^−1^, MnCl_2_· 2H_2_O, 1.81 mg L^−1^, ZnSO_4_· 7H_2_O, 0.222 mg L^−1^, Na_2_MoO_4_· 2H_2_O, 0.39 mg L^−1^,CuSO_4_· 5H_2_O, 0.08 mg L^−1^, CoCl_2_· 6H_2_O, 0.01 mg L^−1^) (Stanier et al., 1971). The N-deficient medium (N−) was BG11 without NaNO_3_. *Chlorella* sp. A2 was cultured as previously described by Zhang et al. (2013).

### Experimental design of minimal nitrogen supply cultivation

#### Laboratory cultivation

For the laboratory cultivation, cells at the mid-logarithmic growth phase (OD_680_ approximately 0.8) were harvested, washed and resuspended in regular BG11 as the control or N− medium to OD_680_ 0.2. Minimal urea was added at a range of concentrations and at various time intervals to algal cultures with N− medium via two modes. *Chlorella* sp. A2 was cultured in a 500-mL Erlenmeyer flask containing 300 mL medium at 30°C with continuous illumination of 50 μmol photon m^−2^ s^−1^ and continuously bubbled with filtered air.

In the first mode, minimal urea was added daily at the following concentrations: 4.5 mg L^−1^, 9 mg L^−1^, 18 mg L^−1^ and 9 × Int(OD_680_/2.5 + 1) mg L^−1^, respectively. The culture to which 9 × Int(OD_680_/2.5 + 1) mg L^−1^ urea was added had 9 mg/L urea added daily if OD_680_ was below 2.5, and an additional 9 mg L^−1^ urea for every increase in OD_680_ 2.5.

In the second mode, 18 mg L^−1^ urea was added at various time intervals. The corresponding time intervals were once a day, every 2 days, every 3 days, or every 4 days, respectively.

#### Outdoor cultivation

For the outdoor cultivation, cells at the mid-logarithmic growth phase (OD_680_ approximately 0.8) were harvested, washed and resuspended in regular BG11 as the control or N− medium to OD_680_ 0.2. Minimal urea was added at various concentrations or time intervals using the same two modes described above for laboratory cultivation. *Chlorella* sp. A2 was cultured in a 5-L photobioreactor containing 4 L medium at outdoor temperature with natural lighting, and filtered air was continuously bubbled through. On cloudy or rainy days, the fill lights were applied to maintain the illuminance at more than 6000 lux.

### Biomass growth analysis

To check the growth of the cells, the absorbance of each culture was read at 680 nm in a UV-1800PC spectrophotometer, and a corresponding blank medium without algae was used as the control. To check the cells' dry weight, they were harvested by centrifugation, dried using a freeze dryer and weighed.

Growth rate was calculated by the formula, μ = Ln (N_2_/N_1_)/(t_2_–t_1_), and biomass productivity was calculated by the formula, biomass productivity (g L^−1^ d^−1^) = (N_2_–N_1_)/(t_2_–t_1_), where N_1_ and N_2_ are defined as the biomass at time 1 (t_1_) and time 2 (t_2_), respectively.

The actual urea consumption per unit biomass growth was calculated by the formula, U = (u_0_ + u_1_ + ⋯ + u_i_)/(V·ΔOD_680_), where u_i_ (mg) represents the quantity of urea addition in the i^th^ day, V (L) represents the volume of microalgae cultivation, ΔOD_680_ represents the optical density at wavelength 680 nm of the microalgae on the i^th^ day subtract the value on the 0^th^ day, and U represents the average actual urea consumption per cultivation volume per OD_680_ microalgae over the whole cultivation period.

### Lipid extraction and analysis

#### Lipid extraction and TLC analysis

For the lab mode, we harvest 1 mL microalgae culture at OD_680_ = 1 by the centrifugation at 8000 rpm for 3 min. The cell pellet was washed with fresh medium and centrifuged again. The cell pellet was resuspended in 400 μL of methanol: chloroform mixture (1:1, v/v). The mixture was shaken for 2 min. Then 120 μL of 1 M potassium chloride in 0.2 M phosphoric acid was added for two-phase separation. The mixture was centrifuged at 12,000 rpm at room temperature for 5 min, and the chloroform phase was transferred to another 1.5 mL EP tube. The chloroform phase was dried in fuming hood. Total lipids were extracted according to Reiser and Somerville (1997) with some modifications.

The residue in the tube was resuspended with 20 μL of chloroform to get the lipid extracts. All the 20 μL of resuspended solution was transferred to TLC (Thin-layer chromatography) silica gel plate, and this step should be repeated for 5 times. All residues in a tube were transferred to the silica gel plate in the same location. 200 μg of the commercial glyceryl trioleate reagent (10 mg reagent was resuspended by 100 μL chloroform, and 2 μL resuspended solution was used) was used as a reference substance for triacylglycerol (TAG). The method of the TLC analysis was optimized according to Reiser and Somerville (1997). TAG was separated by silica gel plates in the eluent which composed of hexane-ethyl and ether (3:1, v/v). Lipid samples were visualized by the iodine vapor at 37°C for ~5 min. Then a camera was used to capture the staining pictures. ImageJ (version 1.48, National Institutes of Health, Bethesda, MD) was used to quantify the neutral lipid content in the pictures and calculated as a percentage of cell solution volume. The neutral lipid productivity was calculated by the formula, neutral lipid productivity (g L^−1^ d^−1^) = biomass productivity × neutral lipid content (%).

#### Lipid content determination for algae cultivated in the outdoor environment

The 4 L scale of microalgae cultivation is performed to practice the microalgae industrial production. The microalgae cultivated in the outdoor environment were harvested in order to extract the lipid using the Soxhlet extractor. 500 ml of microalgae culture was sampled for centrifuging at 8000 rpm for 3 min. The cell pellet was washed and then dried. Then the dried cell pellet was weighed for G_0_ and placed into the Soxhlet extractor. Methanol and chloroform (2:1, v/v) was added and the extractor applied at 90°C for 6 h. The solvent mix was dried to achieve constant weight and the residue was weighed for G_1_. The lipid content was calculated by the formula, lipid content (%) = G_1_/G_0_ × 100%. The lipid productivity was calculated by the formula, lipid productivity (g L^−1^ d^−1^) = biomass productivity × lipid content (%).

### Fluorescence microscope analysis

Microscopic analysis of the cells was carried out using a fluorescence microscope (OLYMPUS system microscope BX53, Japan). The transmission micrographs that were used for the visualization of the non-fluorescent protoplast structures were generated using the manufacturer's filter settings. A lipophilic fluorescent dye, Bodipy 505/515 (4,4-difluoro-1,3,5,7-tetramethyl-4-bora-3a, 4a-diaza-sindacene; Invitrogen Molecular Probes, Carlsbad, CA, USA) was used to stain the intracellular oil-containing organelles, known as lipid bodies, with a final labeling concentration of 1 mM and 0.1% DMSO (v/v), according to Cooper et al. (2010). Bodipy fluorescence (green) was excited with an argon laser (488 nm) and measured at 505–515 nm. Autofluorescence (red) of algal chloroplasts was measured simultaneously at 650–700 nm.

### Photosynthetic analysis

#### Quantification of pigments

One hundred percent methanol was used to extract the pigments. The concentrations were spectrophotometrically determined and calculated using the formula developed by Lichtenthaler (1987), as described in Zhang et al. (2013). Each sample was taken for 1 mL with two copies, and one is for OD_680_ detection. Another one was centrifuged at 6000 g at room temperature for 3 min. The cell pellet was resuspended with 1 mL methanol. The pigments extract liquor was stored at 4°C without light over 12 h. Then the spectrophotometer was used to detect the optical density of the pigments extract liquor at 470, 652.4, and 665.2 nm. To compare the distance of the chlorophyll content in biomass level, we developed the original equation and calculated the chlorophyll content per OD_680_.

$$\begin{array}{l} \text{Chlorophyll }a,\text{ Chl }a\\ \;=\;(16.72\;\times \;{\text{OD}}_{665.2}\;-\;9.16\;\times \;{\text{OD}}_{652.4})/{\text{OD}}_{680}\\ \text{Chlorophyll }b,\text{ Chl }b\\ \;=\;(34.09\;\times \;{\text{OD}}_{652.4}\;-\;15.28\;\times \;{\text{OD}}_{665.2})/{\text{OD}}_{680}\\ \text{Total chlorophylls},\text{ Chl }a\;+\;b\\ \;=(1.44\times {\text{OD}}_{665.2}\;+\;24.93\;\times \;{\text{OD}}_{652.4})/{\text{OD}}_{680}\\ \text{Total carotenoids},\text{ Car =}[(\text{1000 }\times \;{\text{OD}}_{\text{470}}\\ \;-\;1.63\;\times \text{ Chl }a\;-\;104.96\;\times \text{ Chl }b)/221]/{\text{OD}}_{680} \end{array}$$

#### Chlorophyll fluorescence analysis

Chlorophyll fluorescence, i.e., Fv/Fm, Φ_II_, and NPQ was measured as described by Zhang et al. (2013) and Kramer et al. (2004). Dual-PAM-100 Chl fluorescence photosynthesis analyzer (Walz, Germany) was used to measure the chlorophyll fluorescence. 3 mL algal samples were dark-adapted for 15 min before measured. The maximum quantum yields of PSII electron transport was calculated as Fv/Fm = (Fm–Fo)/Fm, according to Kitajima and Butler (1975). The effective quantum yields of PSII electron transport was calculated as, Φ_II_ = (Fm'–F)/Fm', according to Genty et al. (1989) and Kramer et al. (2004). The non-photosynthesis quenching of PSII was calculated as NPQ = Fm/Fm'–1, according to Bilger and Björkman (1990).

### Statistical analysis

Each result shown is the mean of at least three biological replicates. The statistical analyses of the data were performed using the program SPSS-13, and the significance was determined at the 95 or 99% confidence limits.*t*-test was used to determine the means and SD of replicated studies. The significant differences between the control and test values were tested by using one-way ANOVA test, and differences were considered to be significant at *P* < 0.05 or *P* < 0.01.

## Results

### Addition of minimal urea has no significant effect on the biomass growth of *Chlorella* sp. A2

**Figure 1:**
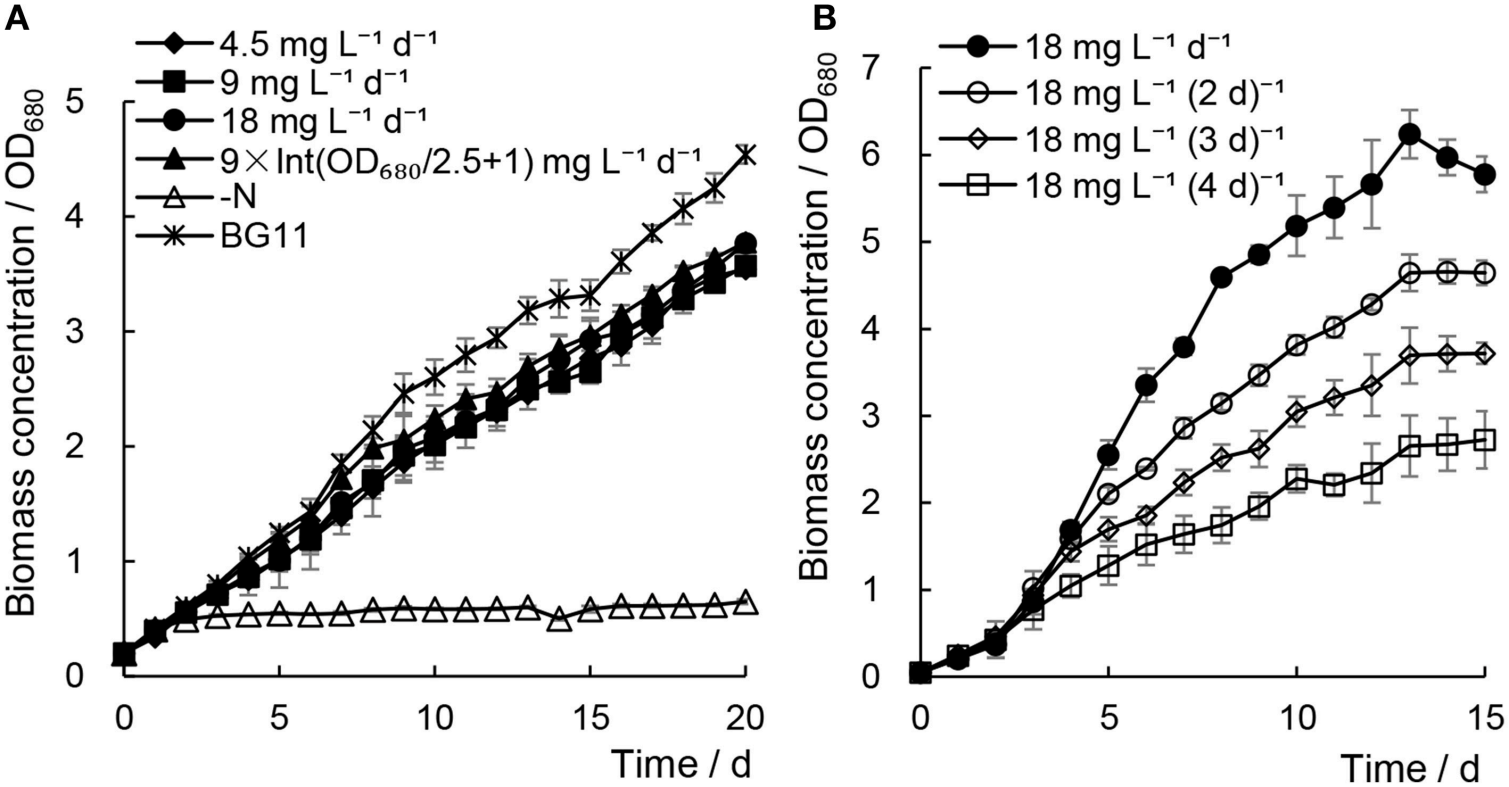
**Biomass growth of *Chlorella* sp. A2 cultivated with minimal urea**. All data points in the current and following figures and tables represent the means of three replicated studies in each independent culture, with the SD of the means (*t*-test, *P* < 0.05, or *P* < 0.01), and the significance of the differences between the control and other test values was tested using a one-way ANOVA at the 95 or 99% confidence limits.

When addition of minimal urea was carried out every day, the biomass growth of *Chlorella* sp. A2 showed much faster cell growth rates (e.g., 0.172–0.180 at 15^th^ days) compared with the N− culture (e.g., 0.071 at 15^th^ days) (Figure 1A). In order to compare with the cell growth and lipid accumulation in *Chlorella* cultured under traditional culture conditions, *Chlorella* sp. A2 was cultured in regular BG11 medium as the control. Although growth rates were decreased slightly (e.g., 0.180 decreased to 0.172 at 15^th^ days) with decreasing concentrations of minimal urea (18 decreased to 4.5 mg L^−1^ d^−1^), the biomass growth did not differ significantly from that of the BG11 control (e.g., 0.187 at 15^th^ days) (Figure 1A). Of the cultures to which minimal urea was added, the best growth was detected in cultures with urea supply at 9 × Int(OD_680_/2.5 + 1) and 18 mg L^−1^ d^−1^. In general, the urea consumption was 100.98 ± 2.63 and 65.46 ± 3.39 mg L^−1^ ${\text{OD}}_{680}^{-1}$ for the algae cultivated with 18 mg L^−1^ d^−1^ and 9 × Int(OD_680_/2.5 + 1), respectively.

As shown in Figure 1B, when 18 mg L^−1^ urea was added at various time intervals, the growth rate of the microalgae declined with increased time intervals; we suggest this was because the longer time intervals may cause a nitrogen-starvation status for microalgae. We concluded that cultivation of *Chlorella* sp. A2 with the addition of 18 mg L^−1^ d^−1^ urea is an appropriate strategy for optimal biomass growth.

### Photosynthetic capacity was not adversely affected by minimal nitrogen supply

**Figure 2:**
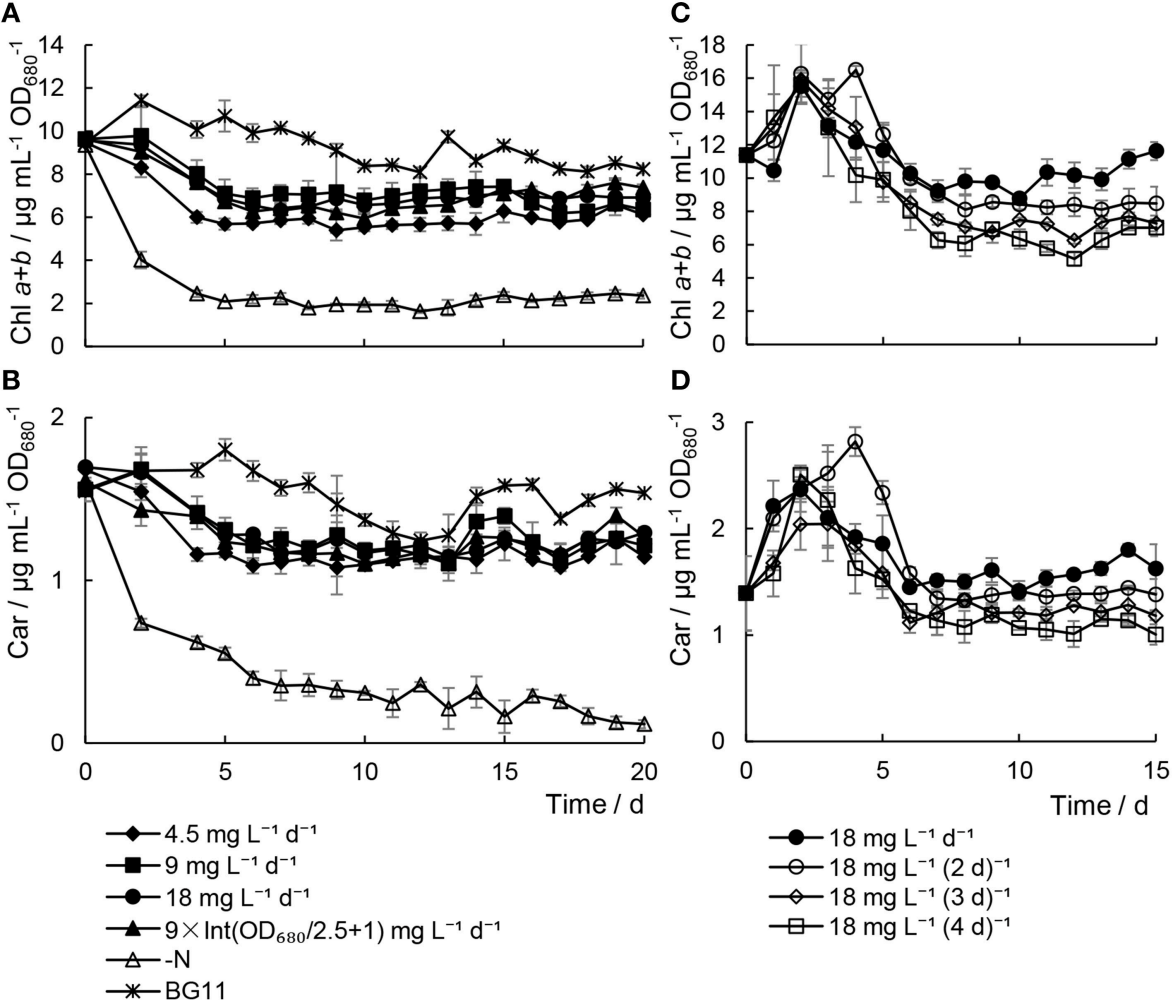
**Variations in pigment contents in *Chlorella* sp. A2 cultivated with minimal urea. (A,B)** Shows pigments content in the mode of different concentration urea addition daily; and **(C,D)**, in the mode of urea addition at different time intervals. **(A,C)** Shows the content of chl *a*+*b*; **(B,D)**, car (carotenoid).

In the *Chlorella* sp. A2 cultures with minimal nitrogen, the content of photosynthetic pigments was slightly lower than that of the cultures in BG11 media (Figures 2A,B, Figure S1), which indicated that insufficient urea concentration had no significant damaging effect on the photosynthetic pigments in *Chlorella* sp. A2. In contrast, there were significant decreases in the photosynthetic pigment content in cells in N− medium (one-way ANOVA test between cells with minimal nitrogen and N− medium, *P* < 0.01). In addition, in the microalgae cultivated with minimal nitrogen at a range of time intervals, the microalgae cultivated with 18 mg L^−1^ d^−1^ urea addition had the highest level of photosynthetic pigments (Figures 2C,D, Figure S1).

**Figure 3:**
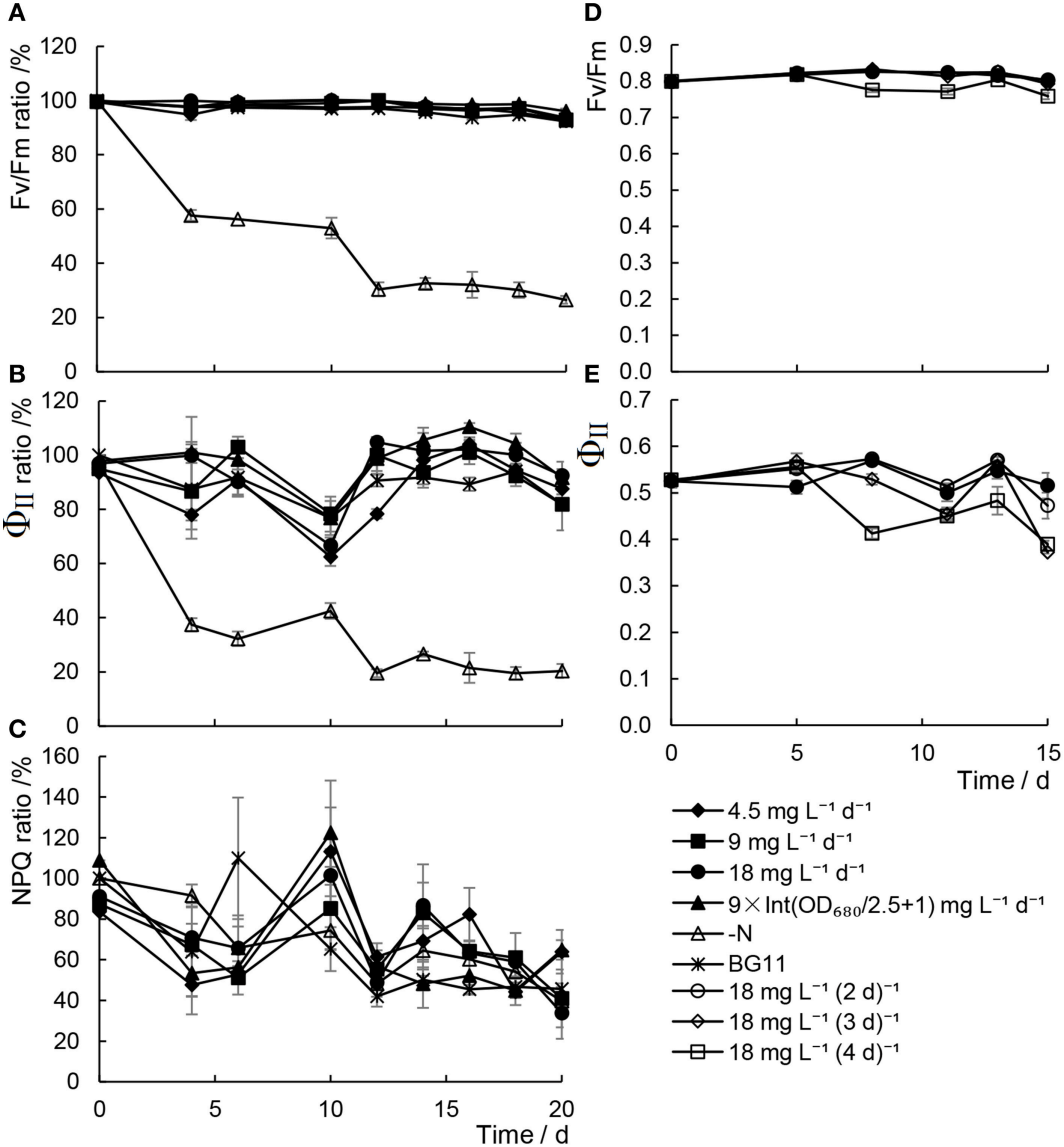
**Chlorophyll fluorescence parameters of *Chlorella* sp. A2 cultivated with minimal urea. (A–C)** Show chlorophyll fluorescence parameters in the mode of different concentration urea addition daily; and **(D,E)**, in the mode of urea addition at different time intervals. **(A–C)** Shows the ratio changes of Fv/Fm, Φ_II_ and NPQ, respectively. **(D,E)** Show the value changes of Fv/Fm and Φ_II_, respectively.

Chlorophyll fluorescence has been considered one of the most sensitive and non-invasive tools for investigating the stress responses of photosynthesis under unfavorable conditions (Maxwell and Johnson, 2000). As shown in Figure 3, it was revealed that both the maximum quantum yield of PSII (Fv/Fm) and the effective quantum yields of PSII (Φ_II_) declined remarkably in cells in N− medium. In contrast, although a slight decrease in the levels of Φ_II_ was detected, which was in keeping with the photosynthetic pigments content, Fv/Fm were unaffected by adding scant urea, either daily or at a range of time intervals. These results suggested that although the efficiency of the photosynthesis did decrease slightly with inadequate urea treatments, the photosynthetic capacity, that is, the photosynthetic apparatus, was not damaged under these conditions.

### Minimal nitrogen supply promoted lipid productivity

**Figure 4:**
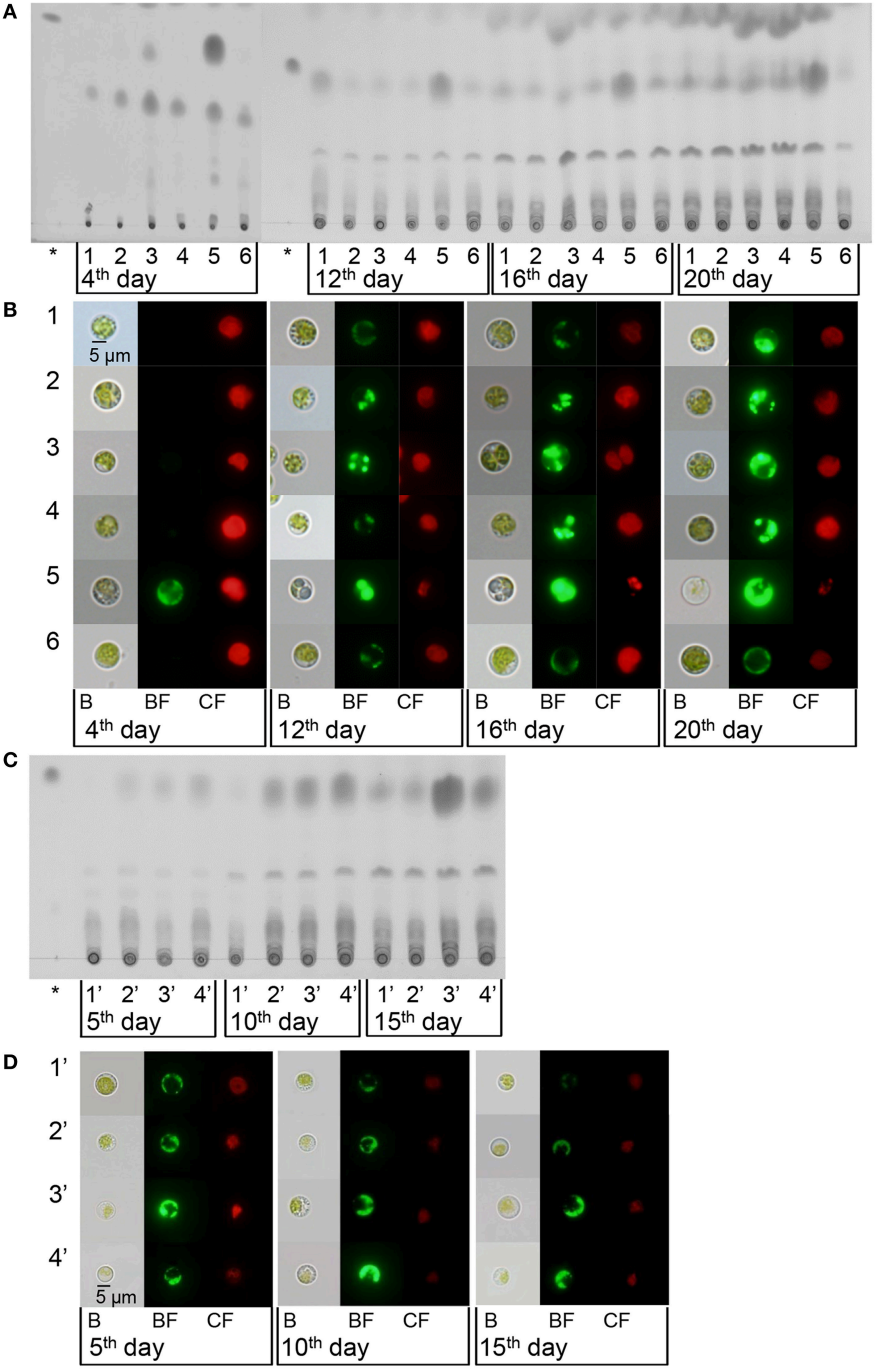
**Lipid accumulation of *Chlorella* sp. A2 cultivated with minimal urea. (A,C)** TLC analysis of neutral lipid accumulation of *Chlorella* sp. A2 with a range of concentrations of minimal urea addition per day **(A)** and with minimal urea addition at different time intervals **(C)**. **(B,D)** Laser scanning micrographs of *Chlorella* sp. A2 labeled *in vivo* with Bodipy 505/515 with a range of concentrations of minimal urea addition per day **(B)** and with minimal urea added at a range of time intervals **(D)**. Bodipy 505/515 (green) was excited with an argon laser (488 nm) and measured at 505–515 nm. Chlorophyll autofluorescence (red) was measured simultaneously at 650–700 nm. The asterisk symbol represents the lipid marker; B, bright field; BF, Bodipy 505/515 fluorescence; CF, chlorophyll fluorescence; 1, 4.5 mg L^−1^ d^−1^; 2, 9 mg L^−1^ d^−1^; 3, 18 mg L^−1^ d^−1^; 4, 9 × Int(OD_680_/2.5 + 1) mg L^−1^ d^−1^; 5, −N; 6, control (BG11); 1′, 18 mg L^−1^ d^−1^; 2′, 18 mg L^−1^ (2 d)^−1^; 3′, 18 mg L^−1^ (3 d)^−1^; 4′, 18 mg L^−1^ (4 d)^−1^. The size of the scale bar is indicated directly on the image.

According to both the TLC (Figure 4A) and the microscopy (Figure 4B), after 12–20 days of treatment, compared with regular BG11 grown cells, the N-starved cells accumulated higher levels of neutral lipids, as expected (Figures 4A,B). Those cells cultured with daily addition of minimal urea accumulated more neutral lipids than in regular BG11 medium and just a slightly lower than in N− medium, which also increased gradually with treatment time. These results indicated that, similar to N starvation, insufficient urea could induce neutral lipid accumulation in *Chlorella* sp. A2, without sacrificing accumulation of biomass (Figure 1A).

Microalgae use as biodiesel feedstock should ideally show high biomass productivity and efficient biosynthesis of lipids, i.e., it is not the single parameter (lipid content or growth rate) but the volumetric lipid productivity that should be the main criterion for choice of feedstock for biodiesel production. With 18 mg L^−1^ d^−1^ and 9 × Int(OD_680_/2.5 + 1) mg L^−1^ d^−1^ urea addition, the microalgae have the highest lipid productivity of 70.57 ± 14.58 and 68.56 ± 13.00 mg L^−1^ total over 20 days (Figure S2A and Table S1A). Compared with the microalgae cultivated in BG11 and N− medium, the algal culture with 18 mg L^−1^ d^−1^ urea addition was able to accumulate ~74 and 416% (w/w) more neutral lipids. Urea addition at other concentrations obtained lower but considerable lipid productivity. On cultivation with 18 mg L^−1^ urea addition at a range of time intervals, the lipid content of the microalgae was also enhanced. Although microalgae with 18 mg L^−1^ (3 d)^−1^ urea addition achieved the highest lipid content, microalgae have the highest lipid productivity with 18 mg L^−1^ d^−1^ urea addition (Figures 4C,D, Figure S2B, and Table S1B). Evidently, minimal nitrogen supply is an appropriate technique for biodiesel production.

### The strategy of minimal urea addition could be applied in outdoor culturing of *Chlorella* sp. A2

**Figure 5:**
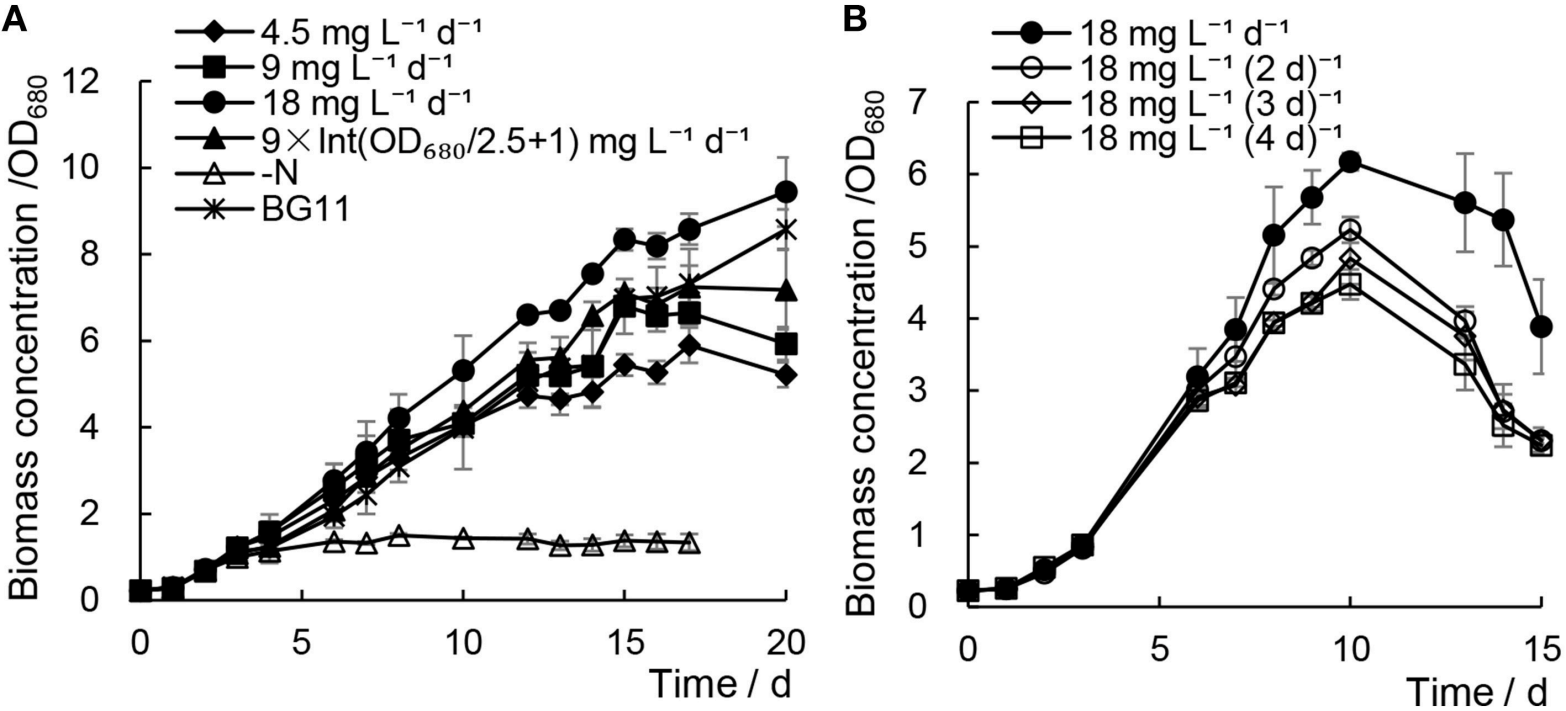
**Biomass growth of *Chlorella* sp. A2 cultivated in the outdoor environment with minimal urea. (A)** Shows biomass concentration changes in the mode of different concentration urea addition daily; **(B)**, in the mode of urea addition at different time intervals.

Biodiesel production will ultimately be practiced in the outdoor environment, so we cultivated *Chlorella* sp. A2 outdoors using the same strategy as above, as illustrated in Figure S3. All the microalgae were cultivated in 5-L conical flasks with the cultivation volume of 4 L. When cultivation is conducted in the outdoor environment, the temperature is affected and controlled by natural condition. As illustrated in Figure 5A and Table 1, the biomass growth of *Chlorella* sp. A2 with the addition of minimal urea also showed much faster cell growth rates compared with those of the N− culture; the microalgae grew fastest and had the highest biomass accumulation when cultivated with 18 mg L^−1^ d^−1^ urea addition, and the growth rate of the microalgae cultivated with 9 × Int(OD_680_/2.5 + 1) mg L^−1^ d^−1^ urea addition was similar to that of microalgae cultivated in the normal BG11 in the prior 16 days. In consideration of the slow cell growth and biomass production of the last 4 days (which arose as the algae reached stationary growth phase), we selected the data in the prior 16 days of cultivation for computing the growth rate, and the results indicated that algae with 18 mg L^−1^ d^−1^ urea addition accumulated biomass with best efficiency (Figure 5A and Table 1). Microalgae with 18 mg L^−1^ urea addition at a range of time intervals were also cultivated in the outdoor environment. Based on the growth rate up until the 10^th^ day, cultivation with 18 mg L^−1^ d^−1^ urea addition was considered appropriate for maintaining the biomass accumulation (Figure 5B and Table 2).

**Table 1:** **Lipid content and lipid productivity of *Chlorella* sp. A2 on 16^th^ day of cultivation**.

**Urea addition**	**Lipid content/%**	**Biomass/g L^−1^**	**Lipid productivity/mg L^−1^ d^−1^**	**Biomass productivity/ g L^−1^ d^−1^**	**Growth rate**
4.5 mg L^−1^ d^−1^	25.19 ± 2.36^A^	1.25 ± 0.06^A^	19.4 ± 0.39^A^	0.078 ± 0.004^A^	0.215 ± 0.003^A^
9 mg L^−1^ d^−1^	23.93 ± 1.28^A^	1.41 ± 0.14^A^	21.3 ± 0.43^B^	0.088 ± 0.009^A^	0.222 ± 0.006^A^
18 mg L^−1^ d^−1^	20.76 ± 0.83^A^	1.66 ± 0.06^A^	21.3 ± 0.46^B^	0.104 ± 0.004^A^	0.233 ± 0.002^A^
9 × Int(OD680/2.5 + 1) mg L^−1^ d^−1^	22.97 ± 2.74^A^	1.57 ± 0.11^A^	22.5 ± 0.50^C^	0.098 ± 0.007^A^	0.229 ± 0.004^A^
– N	38.13 ± 1.66^B^	0.48 ± 0.01^B^	11.3 ± 0.44^D^	0.03 ± 0.001^B^	0.155 ± 0.001^B^
BG11 (control)	21.42 ± 0.24^A^	1.45 ± 0.01^A^	19.4 ± 0.13^A^	0.091 ± 0.001^A^	0.224 ± 0.001^A^

Algae were cultivated in the outdoor environment with daily minimal urea addition.

The significance of the differences between the control and other test values was tested using one-way ANOVA test. A, B, C, and D show significant differences in same item and among groups are represented by different superscripts (P < 0.05).

**Table 2:** **Lipid content of *Chlorella* sp. A2 on 10^th^ day of cultivation in the outdoor environment**.

**Urea addition**	**Lipid content/%**	**Biomass/g L^−1^**	**Lipid productivity/mg L^−1^ d^−1^**	**Biomass productivity/g L^−1^ d^−1^**	**Growth rate**
18 mg L^−1^ d^−1^	22.70 ± 1.13^A^	1.32 ± 0.09^A^	29.91 ± 2.05^A^	0.132 ± 0.009^A^	0.349 ± 0.007^A^
18 mg L^−1^ (2 d)^−1^	24.20 ± 0.57^A^	1.12 ± 0.08^B^	27.02 ± 1.85^A^	0.112 ± 0.008^B^	0.333 ± 0.007^B^
18 mg L^−1^ (3 d)^−1^	24.65 ± 1.12^A^	1.03 ± 0.07^B^	25.32 ± 1.73^A^	0.103 ± 0.007^B^	0.325 ± 0.007^B^
18 mg L^−1^ (4 d)^−1^	19.25 ± 0.21^B^	0.94 ± 0.06^B^	18.06 ± 1.24^B^	0.094 ± 0.006^B^	0.316 ± 0.006^B^

Algae were cultivated with minimal urea at a range of time intervals.

A and B show significant differences in same item and among groups are represented by different superscripts (P < 0.05).

**Figure 6:**
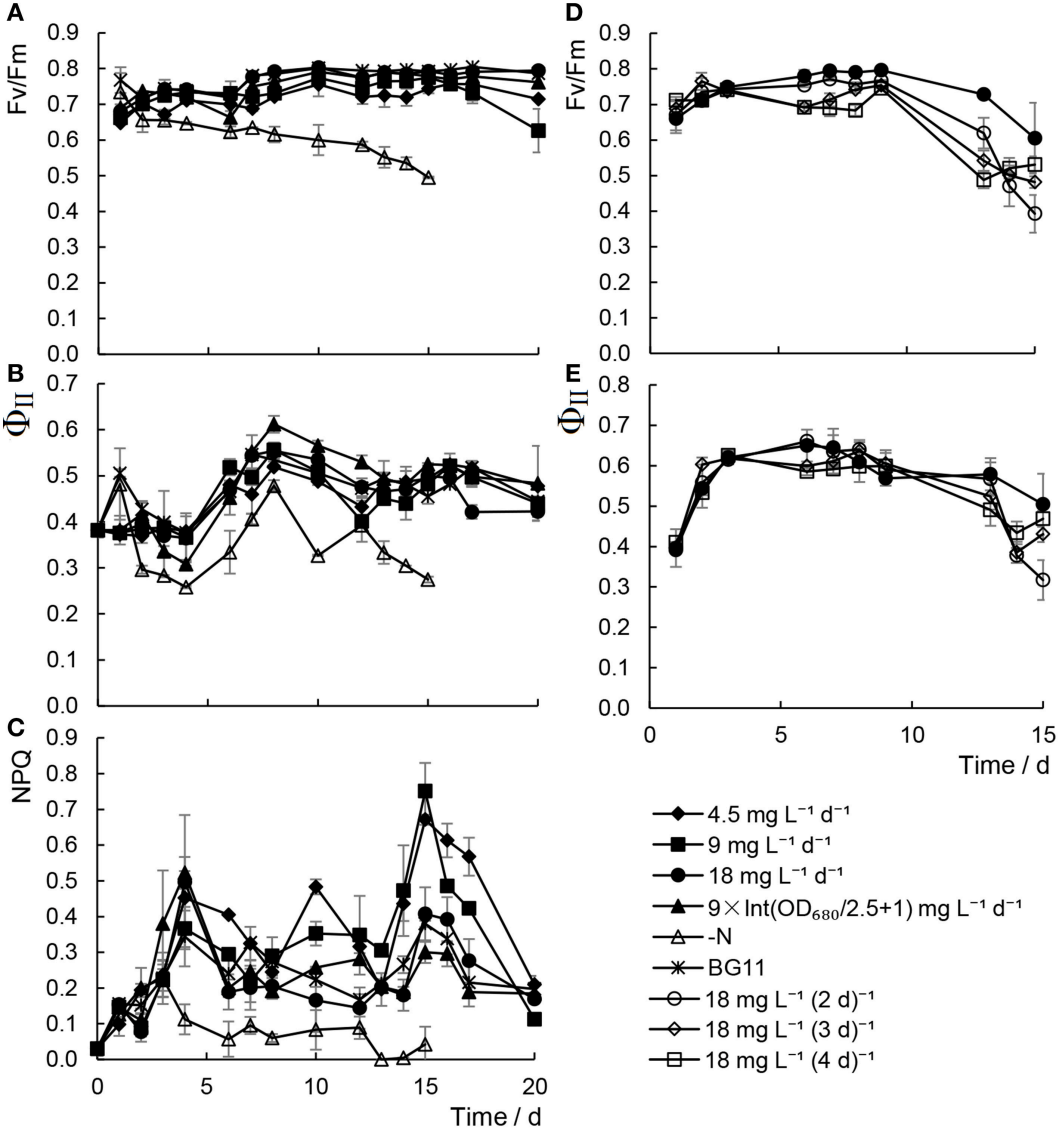
**Chlorophyll fluorescence character of *Chlorella* sp. A2 cultivated in the outdoor environment. (A–C)** Shows chlorophyll fluorescence parameters in the mode of different concentration urea addition daily; and **(D,E)**, in the mode of urea addition at different time intervals. **(A–C)** Shows the value changes of Fv/Fm, Φ_II_, and NPQ, respectively. **(D,E)** Show the value changes of Fv/Fm and Φ_II_, respectively.

We measured the photosynthetic activity parameters of the microalgae cultivated in the outdoor environment: Fv/Fm, Φ_II_, and NPQ. Microalgae cultivated with the minimal nitrogen supply strategy were able to maintain a stable level of Fv/Fm during cultivation, and no significant differences in Fv/Fm, Φ_II_, and NPQ were detected between the regular BG11-grown and the inadequate urea treatments (Figures 6A–E). From the Fv/Fm of the algae cultivated with 18 mg L^−1^ urea addition at a range of time intervals, the 18 mg L^−1^ d^−1^ urea addition was deemed to be an appropriate strategy for maintaining stable photosynthetic activity in the algae.

Still considering the slow cell growth and biomass production of the last 4 days, the lipid production of the algae cultivated in outdoor conditions with daily minimal urea addition was detected on 16^th^ days after cultivation. The lipid content of microalgae cultivated in N− medium was 70% higher than in regular BG11 medium. On the 16^th^ day, microalgae with 18 mg L^−1^ d^−1^ urea addition showed the highest biomass productivity, while the microalgae with 9 × Int(OD_680_/2.5 + 1) mg L^−1^ d^−1^ urea addition showed the highest lipid productivity (Table 1). In cells cultured with 18 mg L^−1^ d^−1^ urea addition, a maximum lipid productivity of 21.3 mg L^−1^ d^−1^ was also much higher (89%) than cells in N− culture. In terms of convenience of operation, 18 mg L^−1^ d^−1^ urea addition may be the best strategy for lipid production. Based on measuring the lipid productivity of microalgae cultivated with 18 mg L^−1^ urea addition at a range of time intervals, on the 10^th^ day there was no significant difference between these algae except algae with 18 mg L^−1^ (4 d)^−1^ urea addition (Table 2). Moreover, 18 mg L^−1^ d^−1^ urea addition with highest lipid productivity was also showed to be the best strategy.

## Discussion

In the course of microalgae biodiesel research, numerous reports have revealed that nitrogen deficiency and limitation could increase the lipid content (Jiang et al., 2012; Liu et al., 2012). Stress can increase the lipid content and can also decrease the biomass, because high lipid content accumulation is often accompanied by weak or inhibited cell division (Courchesne et al., 2009; Gouveia and Oliveira, 2009; Widjaja et al., 2009; Ahmad et al., 2011). While the economic feasibility of microalgae as feedstock for biofuel production depends on three main key factors: biomass accumulation, lipid content, and lipid productivity. Furthermore, biomass and lipid content determine the total lipid productivity. Our strategy did not affect the biomass growth significantly (Table 1).

The minimal nitrogen supply strategy seems to induce the algae, at every daily minimal urea addition, to enter a transient state of lipid accumulation. This may be fundamentally different to the processes occurring in the algal cells under traditional nitrogen-deficiency stress. In the early period of a cycle of 24 h, microalgae tend to undergo cell division and proliferation with the inadequate urea; in the later period, microalgae begin to accumulate more lipids due to the depletion of urea as a result of the nutritional stress. There are two ways that nitrogen-deficiency stress can promote lipid synthesis: one is to compel some carbon fixed during photosynthesis to be channeled into the lipid synthesis pathway (Suen et al., 1987), and another is to cause the carbohydrates to flow into the lipid synthesis pathway directly (Wang et al., 2009; Xin et al., 2010). In addition, in the present study, the photosynthetic capacity was not adversely affected by minimal nitrogen supply. Under conditions of nutritional stress, including nitrogen deficiency, when photosynthesis of algae cells is not interrupted and carbon assimilation is still occurring, carbon flow has been shown to shift from protein synthesis to lipid synthesis (Sheehan et al., 1998).

To lessen the decrease in biomass under conditions of nitrogen deficiency, some researchers invented a two-stage cultivation strategy to improve lipid productivity (Widjaja et al., 2009; Ho et al., 2010), obtaining maximum biomass accumulation in nitrogen-sufficient media and then changing the microalgae conditions to a lipid-accumulation environment. However, the two-stage cultivation strategy is difficult to practice for large-scale cultivation. One approach has also been shown that urea limitation could enhance oil production in other *Chlorella* sp. (Hsieh and Wu, 2009). In the present study, addition of smaller amounts of urea was demonstrated to promote the lipid content and to maintain high biomass accumulation. It appears that, under conditions of low levels of urea addition, a balance between the growth rate and the lipid content accumulation is thus obtained. Compared with the cultivation in regular BG11, this strategy not only unaffected the biomass accumulation of the microalgae, but also promoted the lipid content, and the total lipid productivity was improved (Figure 5A, Table 1). In contrast to the two-stage cultivation strategy, the minimal nitrogen supply strategy can greatly simplify the cultivation process, which has significant advantages in terms of utilizing a single, straightforward cultivation procedure.

In a study by Pirastru et al. (2012), it was found that both PSI and PSII activities were inhibited by nitrogen limitation, and the activity of the PSI declines much more rapidly than PSII. In our previous studies, it was also found that increased damage to PSII was observed in *Chlorella* cells under nitrogen starvation (Zhang et al., 2013; Chen et al., 2015a). However, with increased proportion and energy distribution to PSI in the meantime, the Ca^2+^-regulated cyclic electronic flow increased to produce more ATP for nitrogen starvation-induced lipid synthesis (Zhang et al., 2013; Chen et al., 2014, 2015a). In the present study, only minor differences in Fv/Fm, Φ_II_, and NPQ were detected between the regular BG11-grown and the minimal urea treatments (Figures 6A–E). It is believed that Φ_II_ is well correlated with CO_2_ fixation in the Calvin cycle (Edwards and Baker, 1993). The results indicated that CO_2_ fixation in cells with scant urea treatments is still running regularly, thus providing organic carbon source for constant biomass production (Figures 1, 5, Tables 1, 2).

The application of microalgae in biodiesel production is currently in the laboratory scale and will be expected to just stay in the pilot or exemplary scale in a long time (Chen et al., 2015b). In the present study, cells cultured with addition of minimal urea accumulated 34–44% lower lipids content than in N− medium (Table 1), which means more cells should be harvested and treated for extracting the same lipid yield, and average energy consumption per unit mass of the oil may be higher and extracting lipids from a lower lipid content culture can be more laborious and more costs than extracting from a lipid rich culture. However, the 72–99% increased lipid productivity (Table 1) in those cultures should be more than enough to cover the labor and the energy, and moreover, the lowered lipid content (22%) of cells cultured with addition of minimal urea than in N− medium (38%) is not enough to change the extraction process, i.e., the impact factor of decreased oil content is relatively small in the total cost.

In summary, this study suggests a minimal urea addition strategy for microalgae cultivation for biofuel feedstock production that could promote neutral lipid accumulation without a significant negative effect on cell growth, which is also a single step process and is applicable to outdoor cultivation condition. For achieving excellent biomass growth and lipid productivity, appropriate urea consumption is ~65–100 mg L^−1^ ${\text{OD}}_{680}^{-1}$ for the algae cultivation both in laboratory and outdoor conditions. Notably, our research has clarified the mechanism that minimal urea addition reduced the loss of photosynthetic capacity to keep CO_2_ fixation during photosynthesis for biomass production, and nitrogen limitation promoted the accumulation of neutral lipids, resulting in a significant improvement in the productivity of neutral lipids.

## Author contributions

JZ and WC performed the experiments. JZ, WC, HC, XZ, and CH analyzed the data. QW provided reagents and materials. JZ and WC contributed to the writing of the manuscript and HC revised it. QW and JR conceived and designed the experiments. All of the authors read and final approval of the version to be published.

## Funding

This work was supported jointly by the National Program on Key Basic Research Project (2012CB224803), the National Natural Science Foundation of China (31300030), the Natural Science Foundation of Hubei Province of China (2013CFA109), Sinopec (S213049), and the Knowledge Innovation Program of the Chinese Academy of Sciences (Y35E05).

### Conflict of interest statement

The authors declare that the research was conducted in the absence of any commercial or financial relationships that could be construed as a potential conflict of interest.

## Abbreviations

N−: N-deficient medium
N: nitrogen
N+: N-sufficient medium
OD_680_: the optical density at wavelength 680 nm of the microalgae on the i^th^ day
PSII: the Photosystem II protein complexes
TLC: thin layer chromatography
U: the average actual urea consumption per cultivation volume per OD_680_ microalgae over the whole cultivation period
u_i_ (mg): the quantity of urea addition in the i^th^ day
V (L): the volume of microalgae cultivation.
