# Supplementary material for: Improved Productivity of Neutral Lipids in Chlorella sp. A2 by Minimal Nitrogen Supply
Source: Front Microbiol. 2016 Apr 21;7:557. doi: 10.3389/fmicb.2016.00557 (PMC4838625; doi:10.3389/fmicb.2016.00557)
Supplement: Supplementary file 1 [file Table1.PDF]

Table S1. Quantitation of neutral lipid productivity with minimal urea.

(A). Lipid productivity for microalgae with different concentrations of urea per day.

Time (d)	Neutral lipid productivity (mg L^{-1} d^{-1})					
	4.5 mg L^{-1} d^{-1}	9 mg L^{-1} d^{-1}	18 mg L^{-1} d^{-1}	9 × Int(OD_{680}/2.5 + 1) mg L^{-1} d^{-1}	-N	BG11
4	1.65±0.91^{A}	2.08±1.24^{A}	2.91±1.40^{A}	2.15±1.29^{A}	4.33±0.86^{B}	1.28±0.79^{A}
12	30.64±10.01^{A}	22.52±2.12^{A}	25.82±9.84^{A}	20.16±7.37^{A}	15.76±1.94^{A}	19.44±5.76^{A}
16	44.48±10.22^{A}	49.13±4.84^{A}	57.06±4.42^{A}	47.08±3.46^{A}	12.73±0.78^{B}	37.90±5.39^{A}
20	50.01±6.29^{A}	49.99±8.73^{A}	70.57±14.58^{A}	68.56±13.00^{A}	13.68±1.74^{C}	40.63±2.09^{B}

(B). Lipid productivity for microalgae with 18 mg L^{-1} urea with different time intervals.

Time (d)	Neutral lipid productivity (mg L^{-1} d^{-1})			
	18 mg L^{-1} d^{-1}	18 mg L^{-1} $(2\ d)^{-1}$	18 mg L^{-1} $(3\ d)^{-1}$	18 mg L^{-1} $(4\ d)^{-1}$
5	2.62±0.82^{A}	2.15±0.29^{A}	2.27±0.33^{A}	2.88±0.31^{A}
10	29.96±3.39^{A}	32.17±5.63^{A}	29.49±2.99^{A}	22.11±4.16^{A}
15	58.44±6.83^{A}	52.58±9.89^{A}	50.88±8.74^{A}	38.09±8.09^{B}

All data points in the current and following figures represent the means of three replicated studies in each independent culture, with the SD of the means (*t* test, $P < 0.05$ or $P < 0.01$), and the significance of the differences between the control and other test values was tested using a one-way ANOVA at the 95% or 99% confidence limits. A, B, and C show significant differences in same item (time) and among groups are represented by different superscripts ($P < 0.05$).
