# Supplementary material for: Improved Productivity of Neutral Lipids in Chlorella sp. A2 by Minimal Nitrogen Supply
Source: Front Microbiol. 2016 Apr 21;7:557. doi: 10.3389/fmicb.2016.00557 (PMC4838625; doi:10.3389/fmicb.2016.00557)
Supplement: Supplementary file 2 [file Image1.PDF]

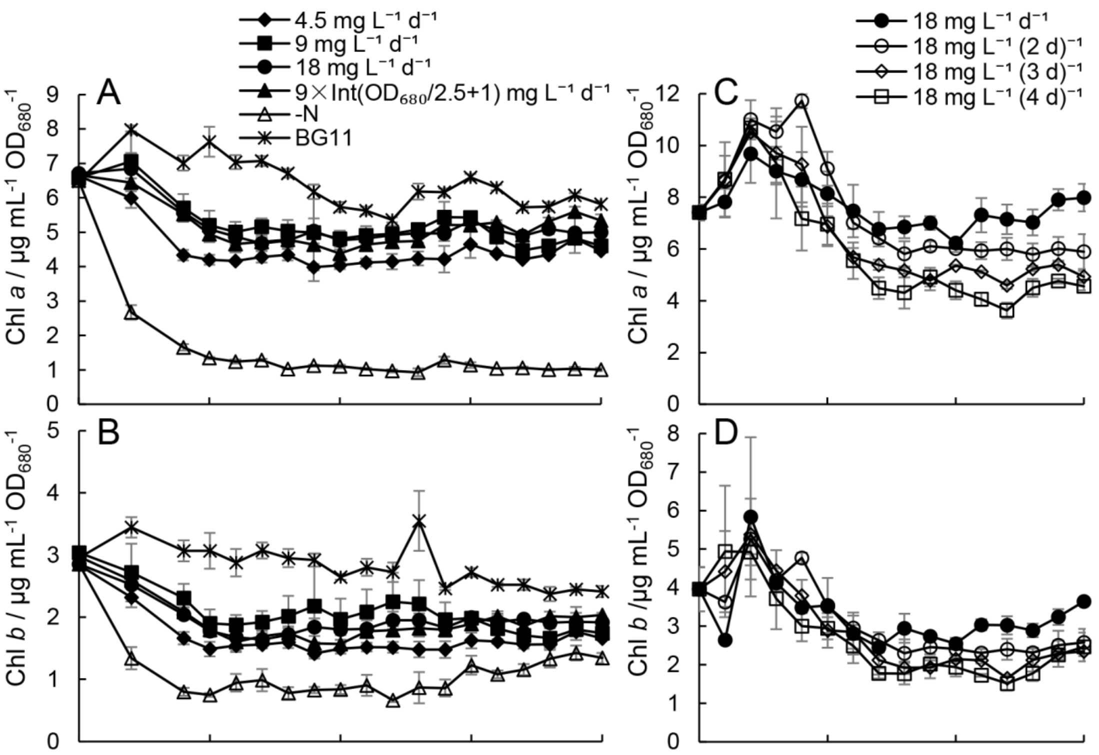

Fig. S1. Variations in pigment contents in *Chlorella* sp. A2 cultivated with minimal urea. (A) and (B) shows pigments content in the mode of different concentration urea addition daily; and (C)–(D), in the mode of urea addition at different time intervals. .(A) and (C) shows the content of Chl a; (B) and (D), Chl b.

.
