# Supplementary material for: Improved Productivity of Neutral Lipids in Chlorella sp. A2 by Minimal Nitrogen Supply
Source: Front Microbiol. 2016 Apr 21;7:557. doi: 10.3389/fmicb.2016.00557 (PMC4838625; doi:10.3389/fmicb.2016.00557)
Supplement: Supplementary file 3 [file Image2.PDF]

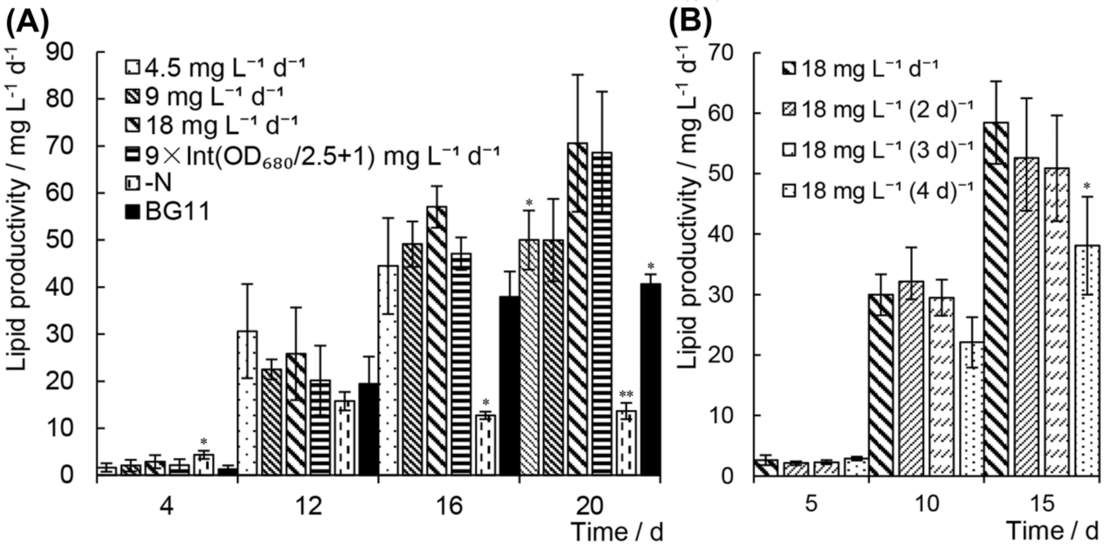

Fig. S2. Lipid productivity of *Chlorella* sp. A2 cultivated in the lab with minimal urea. (A). Neutral lipid productivity for microalgae with different concentrations of urea per day. (B). Neutral lipid productivity for microalgae with 18 mg L^{-1} urea with different time intervals. The significance of the differences between the control (18 mg L^{-1} d^{-1}) and other test values were tested using a one-way analysis of variance, **P*<0.05 vs. control, ***P*<0.01 vs. control.
