# Supplementary material for: Improved Productivity of Neutral Lipids in Chlorella sp. A2 by Minimal Nitrogen Supply
Source: Front Microbiol. 2016 Apr 21;7:557. doi: 10.3389/fmicb.2016.00557 (PMC4838625; doi:10.3389/fmicb.2016.00557)
Supplement: Supplementary file 4 [file Image3.PDF]

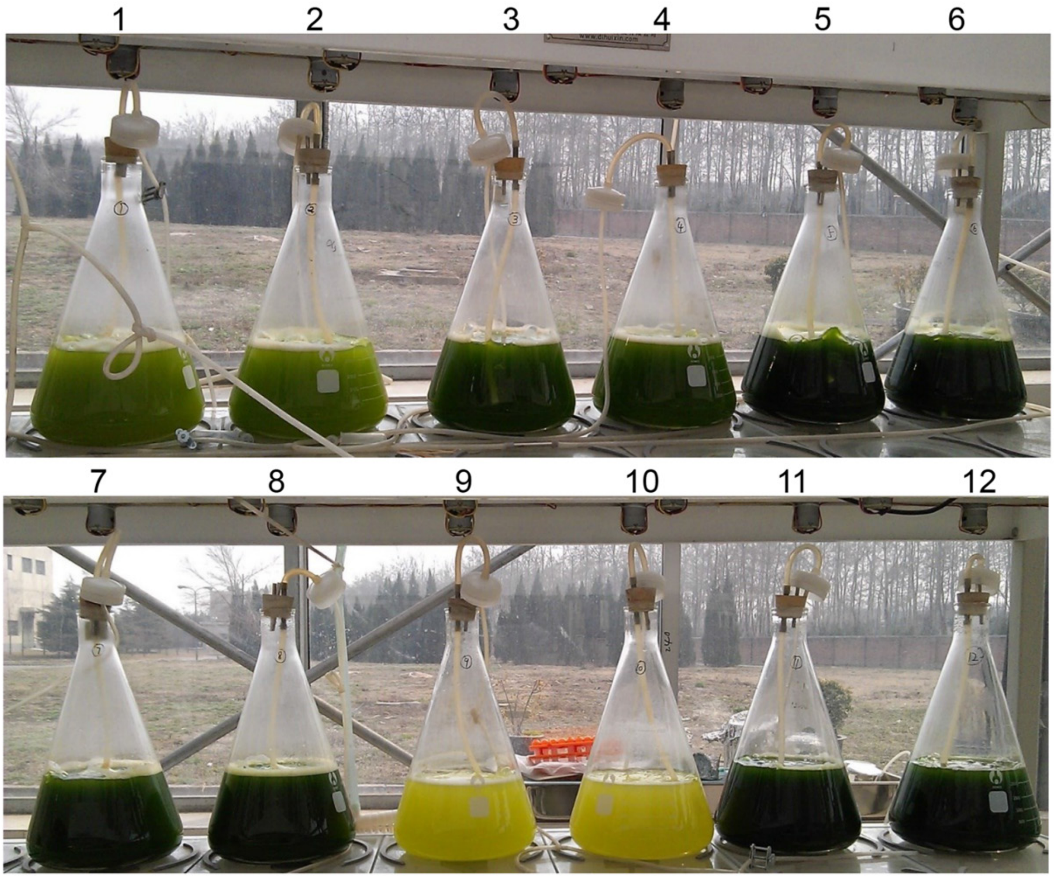

Fig. S3. The outdoor cultivation system for *Chlorella* sp. A2. The number 1 and 2 stand for 4.5 mg L^{-1} d^{-1}; 3 and 4, 9 mg L^{-1} d^{-1}; 5 and 6, 18 mg L^{-1} d^{-1}; 7 and 8, 9 × Int(OD_{680}/2.5 + 1) mg L^{-1} d^{-1}; 9 and 10, -N; 11 and 12, control (BG11).
